# Factors Associated with Pleurisy in Pigs: A Case-Control Analysis of Slaughter Pig Data for England and Wales

**DOI:** 10.1371/journal.pone.0029655

**Published:** 2012-02-17

**Authors:** Henrike C. Jäger, Trevelyan J. McKinley, James L. N. Wood, Gareth P. Pearce, Susanna Williamson, Benjamin Strugnell, Stanley Done, Henrike Habernoll, Andreas Palzer, Alexander W. Tucker

**Affiliations:** 1 Department of Veterinary Medicine, University of Cambridge, Cambridge, United Kingdom; 2 Veterinary Laboratories Agency (AHVLA), Bury St Edmunds, Suffolk, United Kingdom; 3 Veterinary Laboratories Agency (AHVLA), West House, Thirsk, North Yorkshire, United Kingdom; 4 BQP Ltd, Stradbroke Business Centre, Stradbroke, Suffolk, United Kingdom; 5 Ludwig-Maximilians-Universität München, Clinic for Swine, Sonnenstrasse, Oberschleissheim, Germany; Centers for Disease Control and Prevention, United States of America

## Abstract

A case-control investigation was undertaken to determine management and health related factors associated with pleurisy in slaughter pigs in England and Wales.

**Methods:**

The British Pig Executive Pig Health Scheme database of abattoir pathology was used to identify 121 case (>10% prevalence of pleurisy on 3 or more assessment dates in the preceding 24 months) and 121 control units (≤5% prevalence of pleurisy on 3 or more assessment dates in the preceding 24 months). Farm data were collected by postal questionnaire. Data from respondents (70 cases and 51 controls) were analysed using simple logistic regression models with Bonferroni corrections. Limited multivariate analyses were also performed to check the robustness of the overall conclusions.

**Results and Conclusions:**

Management factors associated with increased odds of pleurisy included no all-in all-out pig flow (OR 9.3, 95% confidence interval [CI]: 3.3–29), rearing of pigs with an age difference of >1 month in the same airspace (OR 6.5 [2.8–17]) and repeated mixing (OR 2.2 [1.4–3.8]) or moving (OR 2.2 [1.5–3.4]) of pigs during the rearing phase. Those associated with decreased odds of pleurisy included filling wean-to-finish or grower-to-finish systems with piglets from ≤3 sources (OR 0.18 [0.07–0.41]) compared to farrow-to-finish systems, cleaning and disinfecting of grower (ORs 0.28 [0.13–0.61] and 0.29 [0.13–0.61]) and finisher (ORs 0.24 [0.11–0.51] and 0.2 [0.09–0.44]) accommodation between groups, and extended down time of grower and finisher accommodation (OR 0.84 [0.75–0.93] and 0.86 [0.77–0.94] respectively for each additional day of downtime). This study demonstrated the value of national-level abattoir pathology data collection systems for case control analyses and generated guidance for on-farm interventions to help reduce the prevalence of pleurisy in slaughter pigs.

## Introduction

Pleurisy is defined as inflammation of the pleural membranes, the serosal surfaces of the lung and chest cavity that facilitates smooth inflation of the lung. It is a particular problem in the pig industry [1] and is evident at necropsy or slaughter as fibrinous or fibrous adhesions between the lung lobes (visceral pleurisy) and/or the lungs and chest wall (parietal pleurisy). Interest in the economic and welfare impacts of pleurisy has increased since the high prevalence of this condition in finisher pigs has become apparent [1]. The economic impacts require further investigation, but chronic pleurisy is associated with increased time to slaughter [2]. It also causes problems in abattoirs because carcases require trimming causing extra labour, slower production line speeds, and result in increased waste. Respiratory disease is known to have significant negative impacts on indicators of pig welfare [3].

Pleurisy is a common finding in slaughter pigs in the UK, as evidenced by data from the systematic abattoir pathology recording under the British Pig Executive's (BPEX) Pig Health Scheme (BPHS); data provided to us from 14 abattoirs showed that of 15,237 slaughter consignments between July 2005 and October 2008, 80% were affected by pleurisy. Within these consignments, at the individual pig level 12.5% of 641,763 pigs were affected. Studies in other countries have found similar and even increasing pleurisy prevalence over the last 20 years (Table 1). Pleurisy is a multifactorial syndrome that can be caused by a number of different infections and which is predisposed to by a range of different management factors.

**Table 1:** Pleurisy prevalence, presented as percentage of individual affected pigs, in EU countries.

Country	Period	Prevalence
Belgium	2000	16% [5]
	2009	20.8% [7]
Denmark	1987	14 [9]
	1998	24% [29]
	2000	25% [4]
Netherlands	1990	12% [14]
	2004	22.5% [14]
Norway	1991	41% [12]
Spain	2009	26.8% [8]
UK	1988	16% [1]

Previous studies of management factors associated with pleurisy in pigs have identified some common management factors, as well as some regional differences. The most important risk factors found in previous studies were related to transmission of infections at herd or pig level such as pig density in neighbourhood [4], [5], poor biosecurity [5], increased herd size [6] or number of pigs per pen [7], lack of complete all-in/all-out practice [4], [8], and mixing of pigs in the finishing stage [4]. But whereas Maes (2001) detected a higher prevalence of pleurisy in slaughter pigs in January/February in Belgium, with more severe lesions in March/April, in the Netherlands Elbers (1992) found highest prevalence in June/August.

The presence of antibodies to *Actinobacillus pleuropneumoniae* (APP) is associated with pleurisy either alone [6], [7], [9], [10] or in combination with Porcine Reproductive and Respiratory Syndromevirus (PRRSV) [8]. Also *Mycoplasma hyopneumoniae* (M. hyo) [7], [11], *Mycoplasma hyorhinis* [12] and Swine Influenza virus (SIV) [6] have been shown to be associated with higher frequency of pleurisy. More recently PCV2 has also been suggested to be associated with increased levels of pleurisy [13], and in addition porcine atrophic rhinitis (PAR) has been associated with pleurisy in Denmark [6], [9].

Understanding the health associated factors and clinical signs in live pigs with pleurisy would permit more effective and timely targeting of control measures, since often the disease is only apparent at slaughter. However, work in this area has been limited—coughing and lethargy are considered to be indicative, but not specific for pleurisy, but attempts to identify pigs suffering from pleurisy pre-mortem based on pyrexia and dyspnoea have not been successful [14].

The present analysis focused on management and health-related associative factors for pleurisy and took into account the three main types of slaughter pig production systems relevant in the European Union (farrow-to-finish, wean-to-finish, grow-to-finish). Most previous studies looked at only one [5] or two types of production systems [8], [9]. A case-control analysis was conducted, using retrospective abattoir pathology data collected at national level within the BPHS over the previous two years. Due to the ubiquity of pleurisy in the UK, pig units were defined as cases or controls based on consistently high or low pleurisy prevalence at unit level. One goal was to demonstrate the value of a nation-wide abattoir pathology database in identifying these consistent case and control units since it provided objective data representing around 80% of the farm assurance accredited English and Welsh production base. Herd specific information on management practices and health observations were gathered by a postal questionnaire from units that met the criteria for case or control.

## Materials and Methods

### Selection of target units based on pre-existing abattoir pathology data

The British Pig Executive (BPEX), representing English and Welsh levy paying pig producers, launched the BPHS abattoir pathology monitoring scheme database in 2005 [15]. BPHS is considered a comprehensive representation of the slaughter pig population in England and Wales since it captures data from approximately 75% of all commercial slaughter herds (1036 of a total 1400 herds, based on 2010 data) [16]. For a given consignment of slaughter pigs, each containing from 10 to >200 pigs, assessments are recorded from every second pig on the slaughter-line up to a maximum sample size of 50 pigs per consignment. The scheme operates at the 14 largest pig abattoirs in England and Wales using 37 specialist veterinarian assessors to collect on-line pathology data on 1 to 4 assessment days per month depending on the size of the abattoir. Assessment days rotate ensuring each day of the week is represented allowing every herd to be assessed at least once a quarter. Standardisation of assessment data between abattoirs and assessors is monitored by the scheme and includes regular training and rotation of assessors [15], [16].

Criteria for case and control definitions were developed from this pre-existing database, taking into account the distribution of the data, and aiming to avoid data collected from small sample populations or from producers that recorded highly variable pleurisy prevalence over time. The database was used to identify all producers that had 50 slaughter pigs assessed on at least three occasions in the 24 months prior to October 2008 (778 (56%) producers of a total of approximately 1400 commercial herds) (Table 2). Fifty nine percent of consignments assessed for these producers had at least a 5% prevalence of pleurisy during the 24 month period but the prevalence was highly variable on some units. As such it was felt important to define a case-control measure based on *consistency* of prevalence of pleurisy over time, in order to attempt to separate units with endemic pleurisy problems from those that exhibited more transient occurrences. *Cases* were defined as those that had >10% of pleurisy-affected pigs in each of the three most recent consignments in the 24 month period prior to October 2008, and *controls* were those that had ≤5% of pleurisy-affected pigs in each of the three most recent consignments in that same period. Selection of these cut-offs was based on examining the distribution of the full dataset while attempting to balance study power and maximum discrimination of case and control groups. Indicative sample size calculations were done on the basis of a single factor analysis and indicated that data would be needed from 105 case units and 105 control units to detect statistical significance (p<0.05) of a risk factor found in 20% of the control units that had an odds ratio of 2.5, with a desired study power of 80%.

**Table 2:** The number (%) of herds at each level of the sampling strategy.

Herds (cases and controls)	Number (%)
Commercial slaughter-pig holdings in England and Wales	1400 (100%) [16]
Herds sampled by BPHS scheme (data for 2010)	1036 (74% of 1400) [16]
Herds with 50 pigs sampled by BPHS on at least 3 occasions prior to October 2008	778 (56% of 1400)
Number of eligible cases	121(16% of 778)
Number of eligible controls	306 (39% of 778)
Total number of eligible herds	427 herds (55% of 778; 31% of 1400)
Number of dispatched questionnaires	242 (121 cases, 121 controls)
Number of completed questionnaires	121 (50% of 242; 16% of 778; 9% of 1400)
	51 cases (7% of 778)
	70 controls (9% of 778)
Number of herds included in univariable model	121
Number of herds included in multivariable model	121

The number (%) of herds at each level of the sampling strategy, including the number of eligible case and control herds, as a proportion of the total number of commercial slaughter-pig herds in England and Wales.

### Questionnaire to collect farm-level information

Herd health and management data were gathered by a closed-question postal questionnaire sent to 242 units (121 cases, 121 controls) followed up by telephone liaison with the farm manager and the appropriate private veterinarian. Respondents were not informed of their case/control categorisation in order to minimise selection bias. A pilot questionnaire was validated at three units before dispatch. The questions were composed to ensure clarity for producers and sufficient detail for statistical analysis. An outline of investigated variable factors is presented in Table 3.

**Table 3:** Outline of variables included in a questionnaire addressed to pig farms.

Variable	Levels (if applicable)
Production unit type (and number of sources where applicable)	Farrow-finish/wean-finish/grow-finish
All-in/All-out pig flow	By unit/ building/room/pen
Number of finisher places	value
Distance to next pig unit (km)	value
Experience of senior stockman (years)	value
Ongoing training of stockmen	Yes/No
Accommodation systems (for weaning −30 kg, and 30 kg – slaughter)	Fully slatted/part slatted/straw yards/assisted ventilation
Number of times pigs moved after weaning	value
Number of times pigs mixed after weaning	value
Is airspace shared by pigs of >1 month age gap?	Yes/no
Maximum number of pigs in shared airspace	value
Feeding regime (for 7–30 kg, for 30–50 kg, and for 50 kg – slaughter)	Meal/pellets/wet feed
	Home-mixed/purchased compound/by-product
	Ad libitum/restrict fed
Medication: number at group level	Product/duration/in feed or water/reason
Medication: individual treatments:	Number in past week/reason
Farmer observations of disease (main effect: none, few, many; where an age effect requested this is 7–30 kg & >30 kg; data requested for 2008 & 2007)	Scours (by age)/sneezing (by age)/coughing (by age)/dyspnoea (by age)/meningitis/wasting (by age)/sudden deaths (by age)/porcine dermatitis and nephropathy syndrome (PDNS)/other
Farmer or herd vet knowledge of specific disease status (believed present, confirmed by vet, believed absent, not known)	porcine reproductive and respiratory syndrome (PRRS))/A. pleuropneumoniae (APP)/Glasser's Disease/enzootic pneumonia (EP)/post-weaning multisystemic wasting syndrome (PMWS)
Vaccination of finisher pigs	Absence of any vaccination/EP (one or 2 dose regime)/Porcine circovirus type 2 (PCV2)/PRRS/Glasser's Disease/Ileitis/Other
Post-weaning mortality	Values for 2008, 2007, 2006
Mortality recording system type	Computer/other
Vet health plan in place on unit	Yes/No

Outline of variables included in a questionnaire addressed to pig farms defined as case (pleurisy prevalence consistently >10%) or control (pleurisy prevalence consistently <5%) to seek relationships between pleurisy and production unit type, key indicators of general management, and health observations.

### Processing and statistical analysis of data

Data were stored and manipulated in Microsoft Access and Excel (Microsoft 2007). All statistical analyses were conducted in the R statistical language (R Core Development Team 2008).

The questionnaire was stratified into a series of categories, representing different characteristics of a unit. These were: general farm information (including production type), mortality and productivity, health status, herd environment and herd management. To explore the data in a systematic manner we stratified the variables into two main groups: those that corresponded to farm management characteristics (for which the influence is possibly independent of the disease status), and disease associated factors (those factors that were directly dependent on the disease status of the farm).

It was necessary to re-categorise some of the categorical variables to ensure that there were >5 observations in any level of the factor and also to aid interpretation. Variables having large numbers of missing values (>60) were removed at the outset, as were those categorical variables that had <5 samples in a group and could not be easily re-categorised. Within each group of variables (e.g. management characteristics and disease associated characteristics) the data were screened by applying a simple logistic regression model to each variable in turn, using a chi-squared likelihood ratio test (LRT) [17], and correcting for multiple comparisons using Bonferroni step-down procedures. The extent and distribution of missing values precluded the development of a comprehensive multivariable regression model. However, it was possible to produce a limited multivariable model examining relationships between pleurisy and some of the more important management related factors obtained from the univariate analyses (see results sections for further discussion). Variable selection was conducted using forwards stepwise selection routines and Akaike Information Criterion (AIC) (using the MASS package in R [18]), including only those variables where p = 0.05 or less in the Bonferroni corrected LRT results. Collinearity between variables was assessed by examining the standard errors. As such, in addition to the univariable results we also present some further discussion regarding associations between some of the explanatory variables based on the constrained multivariable models. As a result of the aforementioned limitations, we did not explore interaction effects in this instance. Goodness-of-fit was assessed using the le Cessie-van Houwelingen normal test statistic for the unweighted sum of squared errors [19], [20], as implemented in the “Design” package in R [21]. Discriminatory power was assessed using the Area Under the Receiver Operating Characteristic Curve (AUC), using the “verification” package [22]. Each observation with a standardised Pearson residual of >2 was removed from the final model in turn to check for undue influence due to outliers.

## Results

### Recruitment of respondent farms

Overall there were 126 respondent farms from the original 242 targeted: 51 cases, 70 controls, with 2 questionnaires unusable due to incorrect herd identification. Three had ceased business. Hence the overall usable response rate was 50%. The mean, minimum and maximum pleurisy prevalences across case producers were 29.5%, 12% and 76.7%. Across control producers the mean pleurisy prevalence was 1.6%, ranging from a minimum of 0% to a maximum of 3.3%.

### Management factors

The univariable results for management related risk factor analysis are shown in Table 4. Absence of all-in/all-out (AIAO) pig herd management was an important factor associated with increased pleurisy (OR 9.3) compared to complete AIAO. All-in/all-out by room was similar to no all-in/all-out practice (OR 0.96). Keeping pigs of more than one month age difference in the same airspace was associated with increased pleurisy prevalence (OR 6.5). In addition there was an association between moving and mixing of pigs on farms and higher levels of pleurisy (OR 2.2 and 2.2 per move/mix respectively). Partial slatted flooring for weaners was a strongly associated factor (OR 21.4), but had a very wide confidence interval (3.7–400).

**Table 4:** Analysis of management related factors related to pleurisy in slaughter pigs.

Variable	Adj. LRTp-value	n	Type	Levels	OR	Lower95% CI	Upper95% CI
Herd management	0.00	117	-	AIAO	-	-	-
			-	By room	0.96	0.05	7.2
			-	Mixed	8.2	3.0	24
			-	None	9.3	3.3	29
Shared air	0.00	121	-	False	-	-	-
			-	True	6.5	2.8	17
Number moves (per move)	0.00	119	-	-	2.2	1.5	3.4
Production type	0.00	121	-	Farrow-to-finish	-	-	-
			-	Wean-to-finish	0.10	0.03	0.28
			-	Grow-to-finish	0.45	0.18	1.1
Disinfect between batches	0.00	121	Finisher	False	-	-	-
				True	0.20	0.09	0.44
Downtime (per add. day)	0.00	81	Grower	-	0.84	0.75	0.93
Partial slatted	0.01	80	Weaner	False	-	-	-
				True	21	3.7	400
Number source units	0.01	116	-	0	-	-	-
			-	< = 3	0.18	0.07	0.41
			-	>3	0.69	0.13	4.0
Clean between batches	0.01	121	Finisher	False	-	-	-
				True	0.24	0.11	0.51
Downtime (per add. day)	0.01	83	Finisher	-	0.86	0.77	0.94
Feed origin	0.02	104	Grower	Homemix	-	-	-
				Purchased	0.22	0.09	0.52
Number mixes (per mix)	0.03	120	-	-	2.2	1.4	3.8
Disinfect between batches	0.04	121	Grower	False	-	-	-
				True	0.29	0.13	0.61
Clean between batches	0.04	121	Grower	False	-	-	-
				True	0.28	0.13	0.61

Results of independent logistic regression models fitted to each management variable in turn, showing odds ratios (OR) and 95% confidence intervals for the variables shown to be statistically significant at the 5% level from univariable logistic regression models using likelihood ratio tests (LRT) with Bonferroni adjustments. Continuous and discrete variables are shown with a dash in the “Levels” column, with the OR corresponding to the OR per unit increase; for the categorical variables the OR is relative to the referent level, which is always shown first.

Factors associated with reduced prevalence of pleurisy included wean-to-finish and grow-to-finish production systems compared to farrow-to-finish systems (OR 0.10 and 0.45 respectively), cleaning and disinfection on finishing batches (ORs 0.24 and 0.20 for cleaning and disinfecting respectively), and on grower batches (ORs 0.28 and 0.29 respectively). Also associated was purchasing feed for growers as compared to home-mixing of feed (OR 0.22). Farrow-to-finish production was associated with higher levels of pleurisy than multisite operations that sourced pigs from other breeding units. However, the protective effect became less strong (and statistically insignificant) when these grow-outs sourced from >3 units (ORs 0.18 for ≤3 sources compared to 0.69 for >3 sources). Finally, longer periods of downtime between grower and finisher batches were associated with reduced pleurisy prevalence (ORs 0.84 and 0.86 for each additional day of downtime respectively).

Due to the stratified nature of some of the variables (e.g. grow-to-finish units do not have weaner accommodation), and the within-unit heterogeneity (particularly with regards to some of the accommodation types), it was difficult to design a sensible multivariable model that included all of the variables, such that there were sufficient samples to produce reasonable statistical power. Instead, we restricted attention to some of the more important variables identified in Table 4. Since we needed complete data in order to use stepwise selection, we excluded variables that had more than 5 missing values (leaving 10/15 variables). Then we excluded all batches that had any missing values across these 10 remaining variables (leaving 110 batches). We then fitted a forward stepwise selection model and report the results in Table 5.

**Table 5:** Results from a constrained multiple regression model.

Variable	Type	Level	OR	Lower 95% CI	Upper 95% CI
Clean between batches	Grower	False	-	-	-
		True	0.33	0.11	0.89
Number of moves (per move)	-	-	2.3	1.5	3.8
Shared air	-	False	-	-	-
	-	True	4.0	1.4	12

Results from a constrained multiple regression model fitted to ten variables across 110 batches to further investigate the relationship between management factors and pleurisy in slaughter pigs. Continuous (or discrete) variables are shown with a dash in the “Levels” column, with the OR corresponding to the OR per unit increase; for the categorical variables the OR is relative to the referent level, which is always shown first.

Interestingly, the strongest variable from the univariable analysis (herd management) was the first to be added, and remained in the model until the final step, where it seems that the combination of cleaning between batches (growers), air-space shared by multiple age groups, and number of moves rendered herd management unnecessary to remain in the model. There was a strong association between shared air and herd management (only 2/30 herds with shared air = true practiced AIAO, compared to 57/80 herds with shared air = false), and also between the number of moves and herd management (median of 1 move for AIAO systems and 3 moves for non-AIAO systems). The association with cleaning between batches and herd management was less pronounced. This final model showed no statistically significant lack-of-fit (p = 0.15) and showed a relatively good discriminatory power (AUC = 0.83). Overall, three observations had an absolute standardised Pearson residual of >2 and <2.5, and three more of >2.5. Removing these in turn made negligible difference to the parameter estimates.

### Disease associated factors

Case units had an increased post-weaning mortality, dyspnoea (both<30 kg and >30 kg in weight), coughing (>30 kg) and increased odds of farmer declared positive status for APP. Also, increased frequency of group medication was associated with pleurisy (Table 6).

**Table 6:** Analysis of health related factors related to pleurisy in slaughter pigs.

Variable	Adj. LRT p-value	n	Levels	OR	Lower 95% CI	Upper 95% CI
Mortality 2007	0.00	117	-	1.5	1.3	1.9
APP( farmer or vet declared)	0.00	92	Absent	-	-	-
			Present	8.8	3.4	25
Mortality 2008	0.00	114	-	1.3	1.1	1.6
Mortality 2006	0.00	111	-	1.3	1.1	1.5
Dyspnoea (>30 kg) 2007	0.00	121	Absent	-	-	-
			Present	4.8	2.2	11
Dyspnoea (>30 kg) 2008	0.01	121	Absent	-	-	-
			Present	4.1	1.9	9.0
Cough (>30 kg) 2007	0.03	121	Absent	-	-	-
			Present	4.4	1.8	12
Number of group medications	0.04	117	0	-	-	-
			1–2	3.6	1.5	10
			> = 3	9.6	2.7	40
Cough (>30 kg) 2008	0.05	121	Absent	-	-	-
			Present	4.0	1.7	10.4
Dyspnoea (<30 kg) 2007	0.05	80	Absent	-	-	-
			Present	4.9	1.9	14

Results of independent logistic regression models fitted to each disease associated variable in turn, showing odds ratios (OR) and 95% confidence intervals for the variables shown to be statistically significant at the 5% level from likelihood ratio tests (p-value) with Bonferroni adjustments. Continuous (or discrete) variables are shown with a dash in the “Levels” column, with the OR corresponding to the OR per unit increase; for the categorical variables the OR is relative to the referent level, which is always shown first.

**Figure 1:**
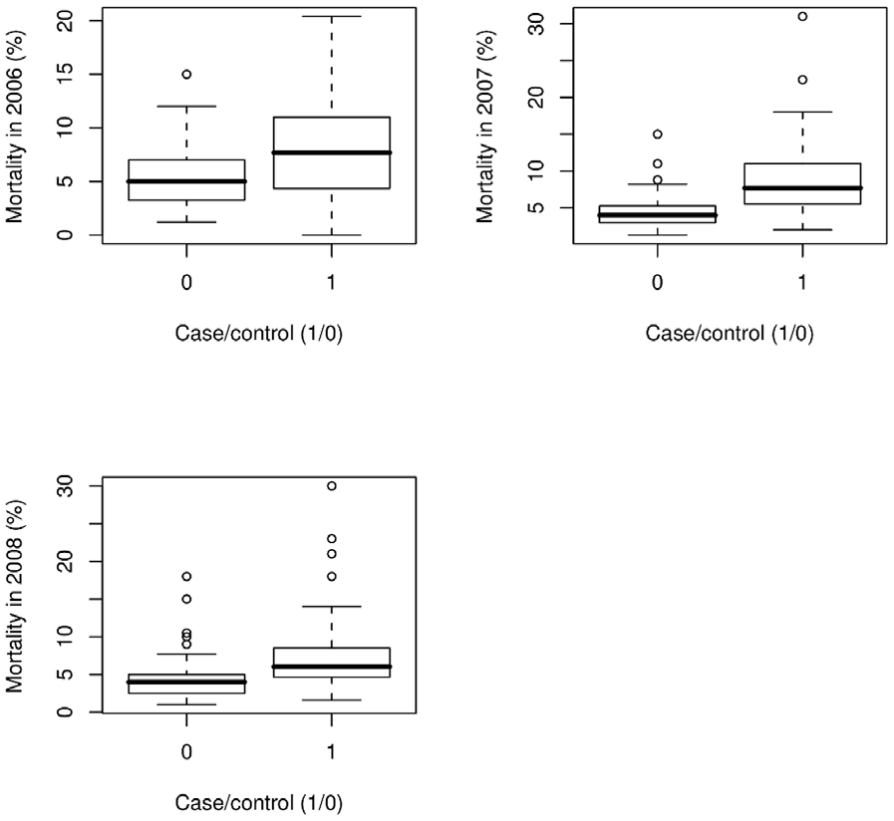
Post-weaning mortality distributions, shown as percentages, for pig farms categorised as pleurisy affected (case) or less affected (control) for 2006–2008.

The median post-weaning mortality rate between 2006 and 2008 (Figure 1) was consistently higher in case units (by 3.3%) (2006: case = 7.7%, control = 5%; 2007: case = 7.7%, control = 4%; 2008: case = 6%, control = 4%. All figures are median values).

## Discussion

The BPHS database, which represents approximately 74% of slaughter pig production in England and Wales, proved suitable for the purpose of identifying case and control units. However, many units within it had a large variation in pleurisy prevalence over the 24 month period studied. Because of this we imposed a strict definition of *consistency* in pleurisy levels over time in our case/control definitions. Hartley (1988) made the same observation regarding pleurisy variability and concluded that this was due to disease dynamics and variation in susceptibility of disease influenced by the environment and management. This may also be impacted by differences from batch to batch in sourcing and mixing of pigs that comprise a batch on entry to a given wean- or grow-to-finish system such that the same unit could have a history of highly variable pleurisy prevalence over time. Chance variation in the infections introduced with different pig batches could be important. The case/control definitions used here provided a metric for distinguishing between *consistently* higher or lower risk units, and must be interpreted as such.

Within responding units there were varying degrees of missing data. This was partly to do with unforeseen heterogeneity in management practices. For example, many units used multiple accommodation types, sometimes for different age groups. These relationships were not clear before the study, but meant that it was difficult to stratify these variables in a sensible manner without incorporating missing information (e.g. stratifying accommodation by age group meant that grow-to-finish units would have missing values for weaning-age variables). Furthermore, there was also a tendency for respondents not to complete all questions. These limitations emphasise the importance of designing data capture questionnaires in a way that maximises the collection of relevant data but minimises the potential for missing data.

Since the definition of cases and controls was determined before recruitment, and the classification was unknown to the respondent, this should reduce the impact of selection bias. Nonetheless, more control farms replied than cases (59% and 41% respectively). We were unable to identify any systematic bias in terms of explanatory variables since we had no data from non-responders. However, the differing response rates suggest that there may be a relationship between producers' ‘attitudes’ to communication about this on-farm health issue and the prevalence of pleurisy. Similar future studies should take account of these differing response rates and factor in the need for follow-up phone calls to responders. Finally, the analysis only included units that had 50 pigs assessed (i.e. 100 or more pigs submitted) on each of 3 successive occasions and, although this means that the results might not extrapolate to small-scale producers, it nevertheless provides information about farm management and health characteristics that are associated with consistently high or low levels of pleurisy in larger, more economically significant, units.

We used a series of univariable logistic regression models using a conservative Bonferroni step-down multiple adjustment procedure [23]. One limitation of this approach is that it is difficult to assess the impact of confounding and effect interactions. As such the individual factors obtained from the univariable analyses that were associated with increased or decreased odds of pleurisy must be viewed in terms of providing information about potential foci for control and intervention that could be tested, and are discussed in the context of other studies and/or prior knowledge. Due to the stratified nature of some of the variables, and the degree-of-missing data, it was only possible to fit a multivariable model to a subset of the data to explore limited associations. However, caution must be used in the interpretation of these results, due to the limited scope of the variables included in the analysis. Nonetheless they further highlight the importance of the variables that were also identified in the univariable analysis.

The results of univariable analysis indicated that failure to implement strict AIAO (by unit or building) was strongly associated with increased pleurisy and this was in line with previous studies [4]. In contrast, the final multivariable model contained cleaning between batches (growers), air-space shared by multiple age-groups, and number of moves but not AIAO. Interestingly AIAO remained in the multivariable analysis until the final step of the procedure before dropping out. Cleaning between batches and avoidance of sharing airspace by pigs of different ages, factors that are both present in the final multivariable model, are important contributory elements of effective AIAO management. Not practising AIAO potentially allows diseases to circulate because susceptible pigs are continuously introduced and older pigs can pass on infections to the younger generation [2]. The univariable analysis findings that repeated mixing, moving, the co-existence of pigs of >1 month age difference in the same air space, and failures in cleaning or disinfection were also factors associated with increased pleurisy reinforced the biological relevance of this observation since these are key practical components of an AIAO management system.

Conversely, implementing AIAO by room, as opposed to by building or unit, was associated with increased pleurisy in the univariable analysis. It seems that there is sometimes confusion about the definition of AIAO – a management system that segregates pigs of a defined age span (e.g. 3 weeks) in an airspace that is separate from groups of other aged pigs throughout their life. A key part of AIAO is that the segregated airspace or accommodation is fully emptied before repopulation occurs. AIAO can break disease cycles, but only if the entire population is included in the process. Our data suggested that AIAO by room cannot be regarded as effective AIAO. In most cases, although the situation varies from farm to farm, a room is not separated enough from other pigs to allow calling the process of emptying a room ‘all-out’ or filling a room ‘all-in’.

The odds of pleurisy increased each time pigs were mixed (univariable analysis) or moved (univariable and multivariable models). Moving and mixing are stressors for pigs which may impact on immunity [24], and are opportunities for pathogens such as APP to spread to susceptible pigs [25]. Although identifying the role of specific infections in causing pleurisy was not a central aim of the current work, vet or farmer-declared presence of clinical APP on the farm was associated with higher levels of pleurisy. APP status might have been determined by clinical or serological status. Vaccination against APP might have impacted on the serological status, or masked clinical disease, but vaccination against this organism is very uncommon in England and Wales. The role of APP in pleurisy is supported by several serological studies [6], [7], [8], [9], [10].

A number of previously undescribed protective factors were identified in this analysis. Firstly, cleaning and disinfection of grower and finisher accommodation between batches was identified in the univariable model, with cleaning of grower pens remaining in the final multivariable model. Secondly, increased “down time” between batches for finisher and grower accommodation was identified in the univariable model. These are issues that have previously been identified as important associative factors relating to enteric disease [26] but less so in the context of respiratory disease. Nevertheless, cleaning might be expected to contribute to respiratory health through reduced levels of dust, environmental bacteria and fungal spores. Resting buildings allows complete drying after disinfection and would be expected to optimise killing of important respiratory pathogens. This has been demonstrated in pig transport trailers for PRRSV [27] but studies of total aerobic bacterial counts were unable to show an effect of down time (Amass 2007). This is nevertheless an important area for future investigation since the presence of organic matter can significantly affect environmental survival of respiratory pathogens such as APP (Gottschalk 2006).

Compared to farrow-to-finish (FF) operations, grow-to-finish (GF) but especially wean-to-finish (WF) systems showed lower levels of pleurisy (GF OR = 0.45; WF OR = 0.1) according to the univariable analysis. The continuous presence of breeding and growing pigs on FF units may be responsible for continuous circulation of infections. Strict AIAO production, at building level, on FF units in the UK is extremely unlikely to occur and pigs must progress through what is often a closely located set of buildings. On the other hand, WF and GF units are more suited to strict AIAO, in spite of the fact that their population usually involves the mixing of pigs from different breeding sources. The observed additional protective effect of WF units over GF units is worthy of further investigation. Of potential importance might be the residual colostrally derived passive immunity at mixing during population in WF units. Population (and mixing of sources) on GF units takes place after the decline of passive immunity with, potentially, a consequential increase in the effective population of susceptible pigs. Also, or alternatively, if infections causing pleurisy spread soon after mixing on AIAO WF units, pigs have a longer period until slaughter during which lesions may resolve.

Another apparently protective factor identified in the univariable analysis was sourcing of piglets to WF or GF sites from ≤3 units in comparison to the single sourcing associated with farrow-finish (no external sources). This association was weaker when a batch was sourced from >3 breeding units. The protective effect over FF may be in part a proxy for the management conditions of WF and GF farms, although the reduced protective effect when more than 3 sources are taken is consistent with the notion that an increase in the likelihood of introduction of disease occurs when sourcing piglets from higher numbers of different units. The use of purchased grower feed versus home mixed feed was found to be associated with lower prevalence of pleurisy (OR = 0.2) but the absence of associations relating to feed at the finisher or weaner stages suggests that this finding may be an artefact, or may be correlated to other factors such as production type (home mixing is more common on FF units in the UK) but this could not be ascertained in the current project.

Regarding associations between pleurisy prevalence and disease related factors, the univariable study differentiated clinical signs by age group (< and >30 kg) and year (2007 and 2008). Similar to previous studies where observable respiratory disease in late finishing was associated with the presence of pleurisy [8], the present study found dyspnoea and coughing in pigs >30 kg were associated with pleurisy in 2007 and 2008. In 2007 dyspnoea in pigs <30 kg could also be related to increased pleurisy in slaughter pigs, but this effect was not observed in 2008. However, these clinical observations are not specific for pleurisy and may indicate other, co-existent, respiratory diseases. Previous research has indicated a link between pleurisy prevalence and prevalence of pneumonia [28], but more recent work suggests this relationship may not be straightforward since lesions of pneumonia were negatively associated with pleurisy lesions [5], [10]. Much opportunity remains to understand how pleurisy relates to pneumonia in pigs and how it might be detected ante mortem.

Increased mortality was consistently and strongly associated with the units being defined as cases in each of the 3 years for which data was requested. This basis of this association is worthy of further investigation because, on one hand, it is another indication that pleurisy is a disease of generally lower health status units and, on the other, an indication of the economic consequences of pleurisy on units where it is a consistent problem. As a proxy for the overall health of a unit, increased numbers of group level medication periods in the post-weaning period were associated with units with consistent pleurisy. While this observation would be consistent with a tendency for pleurisy to occur on units of generally lower health status and with higher consequent production costs, it is probable that some of these additional medications would have been a direct consequence of pleurisy.

In conclusion, this study identified management and health related factors associated with pleurisy based on a questionnaire across 121 respondent units producing slaughter pigs and a national abattoir pathology surveillance database – demonstrating the value of this national disease surveillance system. The identified factors were mostly related to transmission of infectious diseases and the analyses highlighted the importance of AIAO but also a group of management factors associated with it. In addition, farrow-finish management systems were shown to be particularly at risk of consistent pleurisy, in part likely due to the difficulty in implementing strict AIAO in these systems in the UK. Since implementation of complete AIAO management, for example at the building or unit level, has significant cost implications a better understanding of the relative importance of specific management factors that contribute to AIAO and which can be implemented in any production system, is of value to the industry.
